# Vibro-Acoustic Sensing of Instrument Interactions as a Potential Source of Texture-Related Information in Robotic Palpation

**DOI:** 10.3390/s23063141

**Published:** 2023-03-15

**Authors:** Thomas Sühn, Nazila Esmaeili, Sandeep Y. Mattepu, Moritz Spiller, Axel Boese, Robin Urrutia, Victor Poblete, Christian Hansen, Christoph H. Lohmann, Alfredo Illanes, Michael Friebe

**Affiliations:** 1 Department of Orthopaedic Surgery, Otto-von-Guericke University Magdeburg, 39120 Magdeburg, Germany; 2 SURAG Medical GmbH, 39118 Magdeburg, Germany; 3 INKA Innovation Laboratory for Image Guided Therapy, Otto-von-Guericke University Magdeburg, 39120 Magdeburg, Germany; 4 Instituto de Acústica, Facultad de Ciencias de la Ingeniería, Universidad Austral de Chile, Valdivia 5111187, Chile; 5 Research Campus STIMULATE, Otto-von-Guericke University Magdeburg, 39106 Magdeburg, Germany; 6 Department of Measurement and Electronics, AGH University of Science and Technology, 30-059 Kraków, Poland; 7 CIB—Center of Innovation and Business Development, FOM University of Applied Sciences, 45127 Essen, Germany

**Keywords:** robot-assisted surgery, machine learning, minimally invasive surgery, haptic information, vibration sensing, surgical data science, surgery augmentation, tissue classification

## Abstract

The direct tactile assessment of surface textures during palpation is an essential component of open surgery that is impeded in minimally invasive and robot-assisted surgery. When indirectly palpating with a surgical instrument, the structural vibrations from this interaction contain tactile information that can be extracted and analysed. This study investigates the influence of the parameters contact angle α and velocity $\vec{v}$ on the vibro-acoustic signals from this indirect palpation. A 7-DOF robotic arm, a standard surgical instrument, and a vibration measurement system were used to palpate three different materials with varying α and $\vec{v}$. The signals were processed based on continuous wavelet transformation. They showed material-specific signatures in the time–frequency domain that retained their general characteristic for varying α and $\vec{v}$. Energy-related and statistical features were extracted, and supervised classification was performed, where the testing data comprised only signals acquired with different palpation parameters than for training data. The classifiers support vector machine and k-nearest neighbours provided 99.67% and 96.00% accuracy for the differentiation of the materials. The results indicate the robustness of the features against variations in the palpation parameters. This is a prerequisite for an application in minimally invasive surgery but needs to be confirmed in realistic experiments with biological tissues.

## 1. Introduction

### 1.1. Haptic Sensing in Robot-Assisted Surgery

Haptic perception is an essential component of the sensory information available to surgeons while performing any type of interventional procedure. As a source of information, it comprises tactile components such as pressure, vibration, or texture along with kinesthetic components related to the sensing of position, movement, and force [1]. In open surgery, it is a prerequisite for a number of crucial tasks such as palpation for the identification of boundaries and the assessment of tissue stiffness or surface texture. It can be even used for pulsation detection to localise covered vessels.

Instead, in minimally invasive surgery (MIS), specially-designed instruments are used to perform surgery through a small incision in the patient’s skin. While it is considered one of the most significant evolutions in surgery, it comes with a natural decrease in the haptic information available to the surgeon [2]. This source of information is even further reduced by the rise of robot-assisted MIS (RMIS) during the last two decades. While RMIS improves surgical practice by means of higher accuracy and dexterity of instrument manipulation [1] it comes with the significant drawback of loss of natural haptic perception. Some studies see the absence of this source of information as partly accountable for a decrease in surgical efficiency and efficacy or even as a cause of complications [3,4,5]. The loss of haptic perception is mostly compensated by visual control and the use of so-called visual-haptic techniques [6,7,8]. However, visual cues cannot replace the quantity of information possible to acquire via haptic feedback from direct tissue contact.

Due to advances in sensor technology in the last decades, extensive research on tactile sensing modalities and haptic feedback has been conducted in recent years [9,10,11,12]. The majority of the proposed approaches aim to integrate different types of transducers into the instrument used in RMIS or to design completely new instruments [13,14,15,16,17,18,19,20,21]. This approach allows to obtain certain tactile parameters with a limited spatial resolution but requires direct physical interaction with the target tissue. The primary drawbacks are challenges related to the design and manufacturing. Besides a limited installation space, the integration of sensors must not come with any compromise of the initial function or quality of the instrument. Further, the integration of electrical, active components potentially imposes sterilisation and bio-compatibility problems [1]. In contrast, the presented study investigates a different sensing approach that does not require any changes in the surgical instrument.

### 1.2. Vibro-Acoustic Sensing of Instrument Interactions

As pointed out already in 1999 by Bholat et al. [22,23], laparoscopic instruments do provide surgeons with limited and distorted haptic information. Instead of direct contact, the instrument acts as a transfer function between the surgeon’s haptic perception and the tissue or object under examination [24]. The indirect contact simply changes the haptic information arriving and, thus, requires interpretation of the texture, shape, and consistency of the tissue in contact with the surgeon. Due to the nature of RMIS, this information is not directly accessible to the surgeon anymore due to the mechanical decoupling of the robotic instrument and the surgeon’s hand on the steering console. However, because it is intrinsic to any interaction between an instrument and tissue, it is still present in RMIS. It is possible to acquire parts of this information using a sensing setup attached to the instrument itself. Vibrations that originate from the instrument–tissue–interactions propagate naturally along the instrument and can be acquired at any surface location using a vibration sensor. Furthermore, the utilisation of these vibrations is plausible from a biological point of view because it mimics the function of specific mechanoreceptors that react to mechanical pressure or vibration as part of the somatosensory system in the skin [25].

The concept to extract relevant information based on the vibro-acoustic sensing of such interactions was first proposed by Illanes et al. in 2018 [26,27]. The former interpretation by the surgeon is replaced by a signal-processing and analysis strategy dependent on the specific procedure and context. The feasibility of the approach for the extraction of guidance information was shown for percutaneous needle interventions [28,29,30] and catheter-based procedures [31,32]. With respect to an application in RMIS, the potential to extract information related to the surface texture of a palpated object or tissue was explored in [33,34,35]. In these studies, the instrument ProGrasp^™^ Forceps of the da Vinci S RMIS system (Intuitive Surgical, Sunnyvale, CA, USA) was used and signals were acquired using a microphone attached to the surface of the instrument housing. It was shown that the vibro-acoustic signals acquired during the palpation of a material surface allow a differentiation of the surfaces palpated. However, a considerable limitation of these studies was the manually performed movement of the instrument in contrast to robotic actuation of the tool. As a consequence, several parameters of the palpation process such as the contact angle between the palpating instrument and tissue or the velocity of the palpation could not be kept entirely constant.

The aim of this work is to investigate the influence of the parameters of contact angel α and palpation velocity $\vec{v}$ on the acquired vibro-acoustic signals originating from a palpation interaction. Previous studies showed that material-specific information can be extracted from these signals in form of features, which allow the differentiation of interacting materials [36]. The second goal of this study was to assess whether changes in α and $\vec{v}$ can have an influence on this capability of differentiation. To be able to investigate different combinations of the parameters and to keep them constant during the interaction, the Franka Emika Panda robotic system (FRANKA EMIKA GmbH, Munich, Germany) [37] was used for the manipulation of the instrument. Further, the complexity of the sensing setup was reduced to a rod-like instrument and the sensor location was shifted to the proximal end of the instrument. Three different materials $M_{1-3}$ with varying surface structures were palpated using three different combinations of α and $\vec{v}$. In the first step, the acquired signals were processed analysed qualitatively in the time–frequency domain. In the second step, characteristic features were extracted from the signals and used for the training of a supervised classification algorithm. Eventually, the influence of the parameters on the classifier’s performance to differentiate the materials $M_{1-3}$ was assessed. The acquired signals showed a material-specific signature in the time–frequency domain. This signature retains its general characteristic for changes in α and $\vec{v}$ within certain limits. Thus, the extracted features allow a supervised classification of palpation signals acquired with different α and $\vec{v}$ within those limits. This robustness is a prerequisite for an application in MIS, where the surgery-specific constraints do not always allow for defined and reproducible palpation parameters.

## 2. Materials and Methods

### 2.1. Experimental Setup

**Figure 1:**
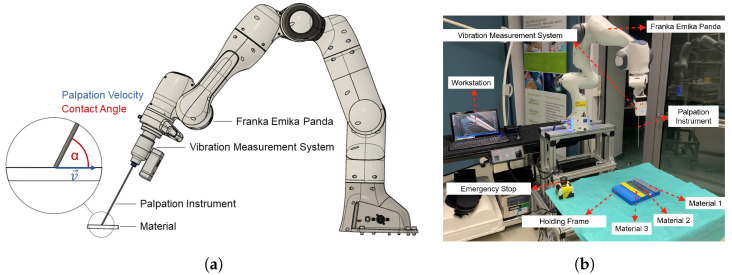
Schematic of the experimental setup (**a**) and implemented setup (**b**) comprising a robotic arm, an instrument with an attached vibration measurement system, and material in contact with palpation parameters ${\vec{v}}_{x}$ and α.

To investigate the influence of α and $\vec{v}$ on the palpation signal as the basis for differentiation materials, the experimental setup depicted in Figure 1a is used. Figure 1b shows the implemented setup comprising a Franka Emika Panda robotic arm, a vibration measurement system along with the connected standard instrument, a holding frame with the materials $M_{1-3}$, the robotic control unit, and the emergency shutdown.

The Franka control interface (FCI) [38] and server version 3.0 was used along with ROS Melodic Morenia [39] to interface the Franka Emika Panda robotic arm and for the development of the control algorithm and interface. As part of the control algorithm, the approaches presented in [40,41,42] were implemented for the computation of the inverse kinematic, subsequent path planning, and provision of a smooth trajectory to the robot control. The implemented control interface allowed for automatic palpation according to specified parameters such as the number of palpations *n*, the velocity of palpation ${\vec{v}}_{x}$, and the contact angle α.

**Figure 2:**
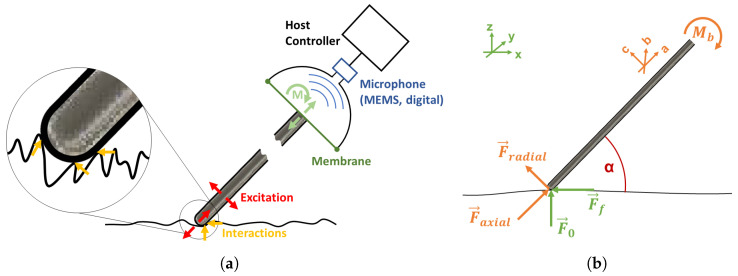
The working principle of the signal acquisition system (**a**) is based on the concept of a stethoscope. A membrane translates the structural vibrations due to instrument interactions to sound waves, which can be acquired with an airborne microphone. The free-body diagram (**b**) visualises the forces and moments originating from the instrument interaction during palpation.

As an indirect palpation (compared to a direct palpation with a surgeon’s hand or finger), we define the interaction of the tip of an instrument with a surface of a material *M* as depicted in the free-body diagram in Figure 2b. The free-body diagram visualises the forces and moments originating from the palpation interaction. We assume a basically plane material surface to be palpated in the coordinate frame (x,y,z). However, this surface is characterised by a specific texture. The tip of an instrument with the coordinate frame (a,b,c) moves with a uniform linear motion of velocity ${\vec{v}}_{x}$ along the surface. During this palpation, the surface texture results in an excitation of the instrument. With respect to the x−y−plane, the instrument was angulated by the contact angles α in the direction of *x* (direction of motion). With respect to *y*, the instrument was orthogonal to the x−y−plane. The Franka Emika Panda robotic system was used for the controlled manipulation of the instrument. This allowed us to keep the parameter ${\vec{v}}_{y}={\vec{v}}_{z}=0$ as well as the *z* coordinate constant during the palpation process and allowed us to only vary the values for α and ${\vec{v}}_{x}$.

In preparation of the data acquisition, the robotic arm was used to gradually reduce the *z* value and to lower the instrument’s tip until it entered into contact with the material. The moment of contact was determined visually by observing the material and instrument tip. Then the robotic arm was lowered an additional 1 mm with the intention to provide a similar indentation depth for all materials and experiments. The indentation of 1 mm is only an approximation that varied slightly for the different materials due to the material properties such as stiffness. These variations of material properties caused different resistance to the indentation and thus indentation depth. Moreover, a slight bending of the rod could not be avoided, which further depends on the used contact angle α. During the palpation, the *z* coordinate was kept constant after the instrument was brought into contact with the material surface.

Due to the indentation, a contact force is acting on the instrument tip over the full palpation path. Because *z* is kept constant, this contact force varies dependent on the characteristic texture of the surface and can be denoted as ${\vec{F}}_{0}\left(x\right)$. Moreover, kinetic friction arises from the interaction of the instrument tip and the material surface during the palpation. This results in a kinetic friction force that is mainly influenced by the kinetic friction coefficient $\mu_{k}$ as well as the contact force ${\vec{F}}_{0}\left(x\right)$ and can be denoted as ${\vec{F}}_{f}\left(x\right)$. The coefficient $\mu_{k}$ is dependent on the properties of the test materials $M_{1-3}$ that vary over the palpation path and the properties of the interacting instrument tip. The interaction forces at the instrument’s tip cause an excitation of the instrument. The resulting structural vibrations propagate along the instrument and can be acquired at the opposite end of the instrument with a vibration transducer. Changes in the interaction forces due to the characteristic surface texture and $\mu_{k}$ result in variations of the excitation and with that the acquired vibro-acoustic signal. As a consequence, each material $M_{1-3}$ should have an individual and distinguishable vibro-acoustic signature over the palpation path.

With respect to the instrument and its coordinate frame (a,b,c), the axial force component ${\vec{F}}_{axial}$ and the radial force component ${\vec{F}}_{radial}$ resulting from the interacting forces are dependent on the contact angle α. Figure 2b depicts the decomposition of the resulting forces into an axial and a radial component ${\vec{F}}_{axial}$ and ${\vec{F}}_{radial}$ as well as the resulting moment $M_{b}$ on the instrument. The moment is dependent on the radial force and the length *r* of the lever, which is the length between the instrument tip and the point of clamping. The influence of α on the force decomposition and resulting moment can be described by Equations (1)–(3).

(1) $${\vec{F}}_{axial}={\vec{F}}_{a}=\sin\alpha \cdot {\vec{F}}_{0}\left(x\right)-\cos\alpha \cdot {\vec{F}}_{f}\left(x\right).$$

(2) $${\vec{F}}_{radial}={\vec{F}}_{c}=\cos\alpha \cdot {\vec{F}}_{0}\left(x\right)+\sin\alpha \cdot {\vec{F}}_{f}\left(x\right).$$

(3) $$M_{b}=r\cdot {\vec{F}}_{radial}.$$

It can be seen that α influences the distribution of axial and radial forces acting on the instrument. Assuming an identical palpation path for one material, differences in α result in different modes of structural vibration of the instrument. Thus, it should result in differences in the acquired signals from the instrument.

### 2.2. Data Acquisition

Compared to the previous study presented in [34], the complexity of the instrument used for the palpation was substantially reduced. An unmodified standard instrument for orthopaedic MIS, known as a palpation probe or changing rod (225-865-027, RZ Medizintechnik GmbH, Tuttlingen, Germany), was used as a basic instrument for the palpation interaction. It is a stainless steel rod with a length of 23 mm, a diameter of 2.7 mm, and a rounded tip that can be used in arthroscopic knee surgery to examine and palpate anatomical structures or to keep the arthroscopic portal open while changing instruments. For the signal acquisition, a wireless vibration measurement system (SURAG Medical GmbH, Magdeburg, Germany) was mounted to the opposite end of the instrument’s tip. It is directly connected to the surface of the instrument via a clamping assembly and does not require any modification of the instrument The system incorporates a mechano-acoustic configuration to acquire structural vibrations from the surface of the instrument with an acoustic transducer. Figure 2a depicts the working principle of the used measurement system that is similar to an extended stethoscope. The instrument is connected to a membrane that translates the structural vibrations due to interactions on the micro- and macro levels to an airborne sound wave. The sound propagates through a confined space to a capacitive, pulse-density modulation (PDM) microphone (SPH0645LM4H-1 Rev A, Knowles Electronics, LLC, Itasca, IL, USA) opposite to the membrane. The microphone is designed as a micro-electromechanical system and comprises an ADC. Subsequently, an analogue signal conditioning a quantisation with a sample frequency of fs=16 kHz and a resolution of 18 bit is performed. The microphone is controlled by a host controller with integrated Wi-Fi capability (Raspberry Pi Zero W, Raspberry Pi Foundation, Cambridge, UK). The acquired signals were stored as audio files in .wav format. The measurement system along with the connected instrument was mounted as an end-effector to the Franka Emika Panda as depicted in the schematic diagram in Figure 1a along with the parameters α and ${\vec{v}}_{x}$ under investigation.

Two different contact angel $\alpha =\{30^{\circ },\;70^{\circ }\}$ were investigated in this study to determine the influence on the acquired vibro-acoustic signal. For the contact angle $\alpha =30^{\circ }$, two different values for the palpation velocity ${\vec{v}}_{x}=\left\{33.3\;\mathrm{mm}/s,\;66.6\;\mathrm{mm}/s\right\}$ were investigated. The first value is based on the velocity achieved during manual palpation by a human subject. During manual palpation, the velocity can hardly be kept stable. Therefore, the second velocity was simply doubled and used to assess the influence of a different velocity on the palpation signal. For the third experiment, the contact angle $\alpha =70^{\circ }$ with a palpation velocity ${\vec{v}}_{x}=66.6\;\mathrm{mm}/s$ was used.

For the data acquisition, a set of three different materials $M=\{M_{1},M_{2},M_{3}\}$ were palpated. The materials were selected due to their substantially different surface characteristics in terms of homogeneity, texture, and irregularities of the surface. Those differences should be reflected in different damping behaviours and interactive forces acting on the instrument, as described earlier. Material $M_{1}$ was industrially produced felt, a non-woven fabric produced by matting, condensing, and pressing of synthetic fibres. It is frequently used in industry as a sound or vibration damper or for polishing. Due to the synthetic fibres, the surface was not outright homogeneous but allowed for a smooth and sliding palpation with the instrument. Material $M_{2}$ was a synthetic studded rubber. In contrast to the level surface of the felt, the small rubber studs caused a slightly bumpy palpation. This resulted in periodic minor impacts and with that excitations of the instrument. Material $M_{3}$ was a dense foamed plastic with a perfectly uniform surface. It caused a similarly smooth palpation as the felt but with increased friction.

The three materials of the size 40 mm × 145 mm were mounted on a holding frame to ensure a stable position during the robot-assisted palpation process. The holding frame comprised two fiducial points that were used to register the position and orientation of the frame to the robot’s coordinate frame. For this, both points were approached with the instrument in vertical orientation until the instrument’s tip was in contact with the holding frame. The dimensions of the holding frame were previously set as fixed parameters in the source code for the palpation. The *z* coordinate was set manually in a way that the indentation of the instrument’s tip was similar for each material. Subsequently, the palpations over a distance of 100 mm could be performed automatically by the robotic arm. A total of n=100 palpation signals were acquired for each of the three materials *M* per experiments 1–3 with the different combinations of α and ${\vec{v}}_{x}$. This resulted in a total of 900 signals for the subsequent analysis. Table 1 summarises the used parameters for the data acquisition in the given experimental setup. Thus, the acquired signals $S_{n}$ were dependent on the parameters $S_{n}\left(M,\alpha ,{\vec{v}}_{x}\right)$. The signal processing steps for the qualitative analysis of the signals are presented in the following section. It forms the basis for the following quantitative analysis.

### 2.3. Signal Analysis

The signal acquisition automatically started with the movement of the robotic arm. As a result, the acquired signals can be separated into two parts. The first part comprises the movement phase of the robotic arm approaching the material surface until the instrument entered into contact. The second part is the actual 100 mm palpation of the material using the defined parameters and involving little movement of the robot. Depending on ${\vec{v}}_{x}$, the physical palpation interaction results in vibration signals of different time lengths obtained by the previously described setup. For the processing, a signal segment of 0.75 s from the middle of each palpation process was used.

The signal can be described by a set of transient responses produced by the tiny interactions between the instrument’s tip and the non-homogeneous surface. The general hypothesis is that the signal contains features that are associated with surface characteristics such as texture or degree of homogeneity. For the analysis and extraction of material-related information, the signals are first segmented into the robot movement phase and the palpation phase. In the first pre-processing step, the palpation signals are band-pass filtered based on the discrete wavelet transform (DWT) using a Daubechies 4 (db4) mother wavelet and with a passband of $f_{p}\approx$ 5–7000 Hz. This step is necessary to detrend the signal and filter high-frequent noise components from the environment. The subsequent processing is based on the continuous wavelet transformation (CWT) using a Morse mother wavelet due to the CWT suitability for signals containing transient responses.

**Figure 3:**
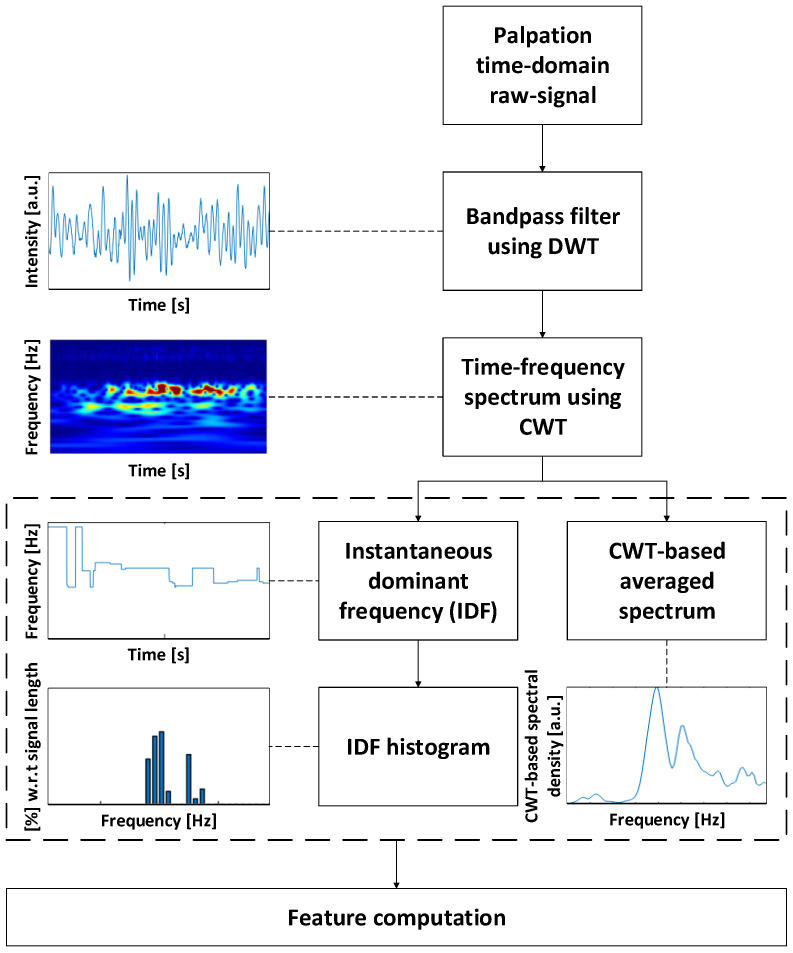
Block diagram of the signal analysis methodology including a bandpass filter with passband $f_{p}\approx$ 5–7000 Hz. The indicators IDF, IDF’s histogram, and averaged spectrum, as well as the CWT spectrum from which they are derived form the basis for the subsequent feature extraction.

After computing the CWT spectrum, three indicators are derived in the time–frequency domains that form the basis for subsequent feature extraction. The block diagram in Figure 3 summarises the processing steps and methods used to compute the indicators. The first indicator is the instantaneous dominant frequency (IDF). It is defined as the particular frequency component dominating the palpation signal at each time instant. The respective frequency in this case is derived from the CWT wavelet scale. For the computation, the frequency involving the maximal spectral energy is calculated for each time instant *t*. It is used as a measure of the stationarity of the palpation process in terms of frequency and is comparable to the pitch in sound perception theory. It potentially allows one to draw conclusions about the homogeneity of the palpated material surface. For example, abrupt changes in the interaction or a set of different dynamics, passing from transient to oscillatory behaviour would cause a frequent change in the IDF.

The second indicator is the probability distribution of the IDF’s values computed as the histogram of the IDF indicator. It represents the distribution of energy in the dominating spectral bands with respect to the full palpation process. Because it is based on the dominating frequency for each point in time, it is less affected by large differences in the energy levels of the interactions. Interactions of small energy that dominate parts of the palpation will equally reflect in the probability distribution as high energy interactions. A corresponding drawback of focusing on the dominant frequency is that the indicator does not reveal the energy distribution of all frequency components present in the signal.

**Figure 4:**
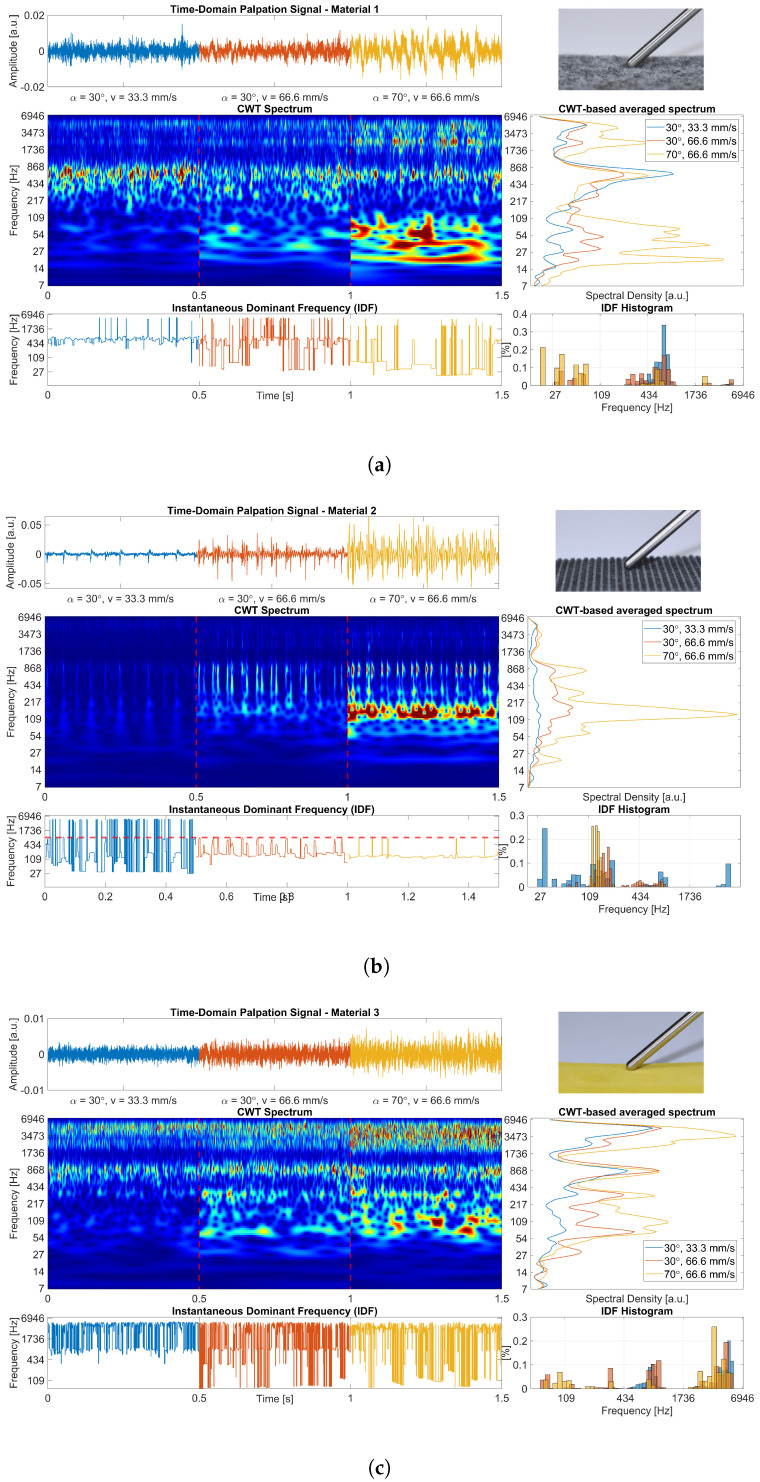
Exemplary palpation signals for Exp. 1 (blue), Exp. 2 (orange), and Exp. 3 (yellow) for the materials $M_{1}$—felt (**a**), $M_{2}$—studded rubber (**b**), $M_{3}$—foamed plastic (**c**). The dashed line (red) in the IDF plot of (**b**) indicates 850 Hz. In the top-right corner, the instrument in contact with the respective material is shown. The CWT-based spectrum and the computed indicators IDF, IDF’s histogram, and stationary spectrum are visualised.

Thus, a third indicator in the frequency domain is defined as the average of the CWT spectrum over time. This allows obtaining a CWT-based stationary spectrum. It represents all frequency components mainly present during one full palpation. Exemplary results of the described signal analysis for the three materials are visualised in Figure 4a–c in the Section 3.1. The generated figures allow for a qualitative evaluation of the palpation signals per material and parameter setting. Further, the characteristic signatures of the individual materials can be visually compared. The signal analysis was performed in MATLAB R2020a. Subsequent to the qualitative analysis, feature extraction and classification of the materials were performed for the quantitative analysis.

### 2.4. Feature Extraction

After the qualitative analysis, scalar features need to be extracted to make the visually perceivable information described in the Section 3.1 accessible and usable for subsequent classification. A total of nine energy-related and four statistical features were extracted from the CWT spectrum or the computed indicators stationary spectrum, IDF and IDF’s histogram. An overview along with a description is shown in Table 2. The nine energy-related features give information concerning the spectral distribution of spectral energies involved in the interaction. Based on observations made from Figure 4, the spectrum was divided into four sub-frequency bands corresponding to pseudo-frequencies (that are derived from the CWT wavelet scale). The sub-bands were determined empirically through extensive visual inspection of a large number of palpation signals. The defined sub-bands span from very low frequencies VLF = 0–116 Hz, low frequencies LF = 116–286 Hz and middle frequencies MF = 286–1300 Hz to high frequencies HF = 1300–7000 Hz.

The first five features were derived from the CWT-based stationary spectrum. Feature 1 was computed as the total energy of the spectrum TE. Features 2–5 were defined as energy proportion corresponding to each of the previously defined bands VLF,LF,MF, and HF. These normalised features $S_{VLF}$, $S_{LF}$, $S_{MF}$, and $S_{HF}$ were obtained by computing the ratios between the energy concentrated inside each band and the total energy TE. In a similar way, features 6–9 were derived from the indicator IDF’s histogram. The same bands were used and $H_{VLF}$, $H_{LF}$, $H_{MF}$, and $H_{HF}$ were computed as the ratios between the IDF’s histogram’s energy in the different bands and the histogram’s total energy.

The four statistical features were related to the frequency components present in the signal. Feature 10 was defined as the frequency of the maximum excitation in the CWT-based spectrum $F_{max}$. It can be thought of as the hot spot in the visual representation of the spectrum as depicted in Figure 4. Features 11–13 are related to the dominant frequencies present in the signal and, thus, are based on the IDF. They are computed as the maximum $DF_{max}$, minimum $DF_{min}$, and variance $DF_{var}$ of the IDF, respectively. $DF_{var}$ for example can be related to the stability or homogeneity of a palpation process.

### 2.5. Classification

Subsequent to the feature extraction, their capability to differentiate the three palpated material surfaces was assessed. This results in a three-class classification problem with $M_{1-3}$ as classes. Two popular supervised classification algorithms were selected for comparison: linear support vector machine (SVM) [43] and k-nearest neighbours (kNN) [44]. The three experiments specified in Table 1 resulted in 3 × 100 palpations per material $M_{1-3}$. This resulted in a dataset with a total of 900 palpations.

The training and testing datasets for the classifiers were compiled following two different approaches. For the first approach, palpation signals from each of the three experiments were included in the training dataset. For that, 70% of the palpation signals of each material per experiment were randomly assigned to the training set. The remaining 30% were assigned to the testing dataset. As a result, in dataset D1 palpation signals from all three experiments are evenly represented in the training and the testing dataset. For the second approach and dataset D2, the training set was formed from the palpation signals from Exp. 1 and Exp. 3 according to Table 1. The two experiments were selected for the training of the classifiers because they represented the highest variety of the palpation parameters α and $\vec{v}$. The idea was to assess if the dynamic of the palpation signals for an individual material could be sufficiently captured by these two experimental conditions. If this hypothesis holds true it would result in a good generalisation of the trained classifiers. Subsequently, differentiation of the materials based on signals acquired with a different combination of parameters α and $\vec{v}$ should be possible. Following this approach, the classifiers were tested against unseen data that was formed by the signals from the remaining Exp. 2. Table 3 summarises the compilation of training and testing data for the two datasets.

In the classification step, the hyperparameter tuning was conducted as part of the training process and separately from the actual testing processes for the given four datasets. Within the training set, 5-fold cross-validation was used and the hyperparameter was optimised using the grid search method [45]. Subsequently, the testing set was applied to assess the performance of the created predictive models. The accuracy, sensitivity, and specificity were calculated from the confusion matrix of each classification scenario as measurement metrics. For the linear SVM, optimisation was conducted to find the regularisation parameter *C*. The range of values for this parameter was set going from 0.001 to 1000. In kNN, the Euclidean distance was used as the distance metric and the number of neighbours *k* was set in the range of 1–1000 with a step size of 1. Table 4 presents the classification results for the different datasets along with parameters *C* and *k* providing the best performances.

## 3. Results

### 3.1. Qualitative Results

Figure 4 shows the time-domain signals along with the computed CWT-based indicators for experiments 1–3 and the materials $M_{1-3}$ (Figure 4a–c). The time-domain signals already allow identifying different signal characteristics for the individual materials as well as differences in the behaviours for the three experiments (per material). However, the CWT-based time–frequency visualisation facilitates a qualitative analysis and a visual comparison of the material-specific signatures. Further, each of the derived indicators facilitates the identification and characterisation of differences between the materials.

The distribution of energy in the CWT spectrum allows observing individual material patterns for particular experiments. These material patterns experience only small changes from one experiment to another. $M_{2}$ for example has its main frequency components between 30 and 1300 Hz while $M_{3}$ has main frequency components between 40 and 1200 Hz but also above 1700 Hz. These components mainly present during one full palpation can be seen in the indicator CWT-based stationary spectrum on the right side of the diagram. It can be observed that the amount of energy and its distribution across the main components present in the signal changes for the experiments. Further, the extent of this change differs depending on whether the palpation velocity (Exp. 1 to Exp. 2) or the palpation angle (Exp. 2 to Exp. 3) was changed. For example, for $M_{1}$, the energy accumulated between 12 and 150 Hz and above 1300 Hz clearly increases in Exp. 3 compared to the first two experiments. Similar behaviour can be observed for $M_{2}$ and the frequency components around 130 Hz. For all materials, the changes between Exp. 1 and Exp. 2 are less prominent and rather appear as a general increase in energy. However, in this representation of the signal it is easy to recognise that the general pattern of each material remains stable from one experiment to another and is distinguishable from the other materials.

In comparison to the CWT-based stationary spectrum, which allows assessing the overall spectral signature of a material, the IDF reveals information about the time-variant spectral behaviour of the signal. The IDF’s behaviour differs considerably in $M_{1-3}$, which confirms the impression of an individual signature per material. In contrast to the stationary spectrum, the IDF’s pattern changes within one material for the different experiments. While the idea of this indicator is to assess the stationarity of the palpation process, it should be not interpreted without the other indicators as can be seen for $M_{2}$. For Exp. 1 and Exp. 2, the periodic transient dynamics at around 850 Hz that can be observed in the CWT spectrum reflect as well partly in the IDF. However, for Exp. 3, the increase in energy of frequency components around 130 Hz leads to a stable IDF of similar frequency. Considering the IDF representation alone for Exp. 3 would hide the mentioned dynamics of 850 Hz, while they are clearly present and recognisable in the CWT spectrum and the CWT-based stationary spectrum. This could potentially lead to a misinterpretation as a stationary palpation process in this particular case.

In general, for the three experiments of $M_{2}$, contrary behaviour of the IDF compared to $M_{1}$ and $M_{3}$ can be noticed. While for the later ones, the variance of the IDF appears to increase from Exp. 1 to Exp. 3, $M_{2}$ shows the opposite behaviour. This reflects as well in the last indicator in the bottom right corner, the probability distribution of IDF values computed as the histogram. It represents the dominating spectral bands with respect to energy distribution. The IDF histograms for the materials are visually easily distinguishable based on the shape and modality of the distribution. For all three experiments per $M_{1-3}$, this indicator confirms the observations obtained from the CWT-based stationary spectrum.

**Figure 5:**
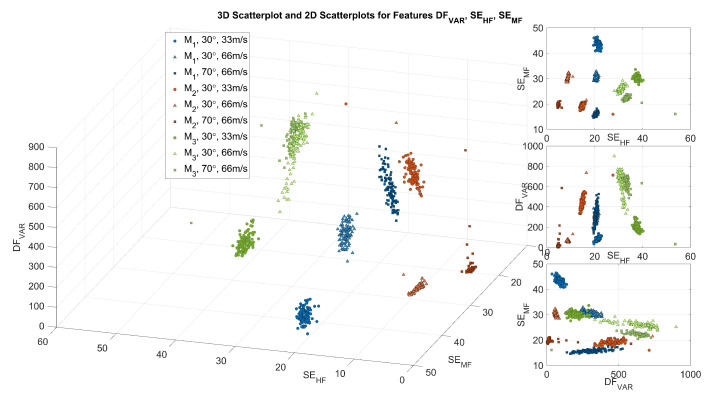
Exemplary 3D scatter plot along with 2D scatter plots of features $DF_{VAR}$, $SE_{HF}$, and $SE_{MF}$ for three palpation experiments per material $M_{1-3}$.

The aforementioned information was exploited to propose the features described in the Section 2.4 and summarised in Table 2. The features should capture parts of the described specific signal characteristic from each material to allow differentiation. Figure 5 shows a three-dimensional scatter plot of the three exemplary features $DF_{var}$, $SE_{HF}$, and $SE_{MF}$ for all signals of Exp. 1–Exp. 3. The features were selected to illustrate the behaviour and to show the capability to form recognisable clusters in the three-dimensional feature space. These clusters are formed based on the individual materials and different parameter setups in Exp. 1–Exp. 3. As an example, the feature $DF_{var}$ confirms an observation previously made in the IDF part of Figure 4. While for $M_{1}$ and $M_{3}$ the variance of IDF values increases from Exp. 1–Exp. 3, $M_{2}$ shows the opposite behaviour. Exp. 1 with $\vec{v}=33.3\;\mathrm{mm}/s$ appears to behave considerably differently in terms of the IDF variance compared to the other experiments for all materials. Further, it can be noticed from $SE_{HF}$ and $SE_{MF}$ that the distribution of energy in the CWT-based stationary spectrum differs for the materials and experiments. After all, it is easy to observe that the exemplary features derived from CWT-based indicators form visually distinguishable clusters, facilitating a classification. To confirm these visual impressions for the given set of features and datasets, two supervised classification algorithms were applied subsequently.

### 3.2. Quantitative Results

**Figure 6:**
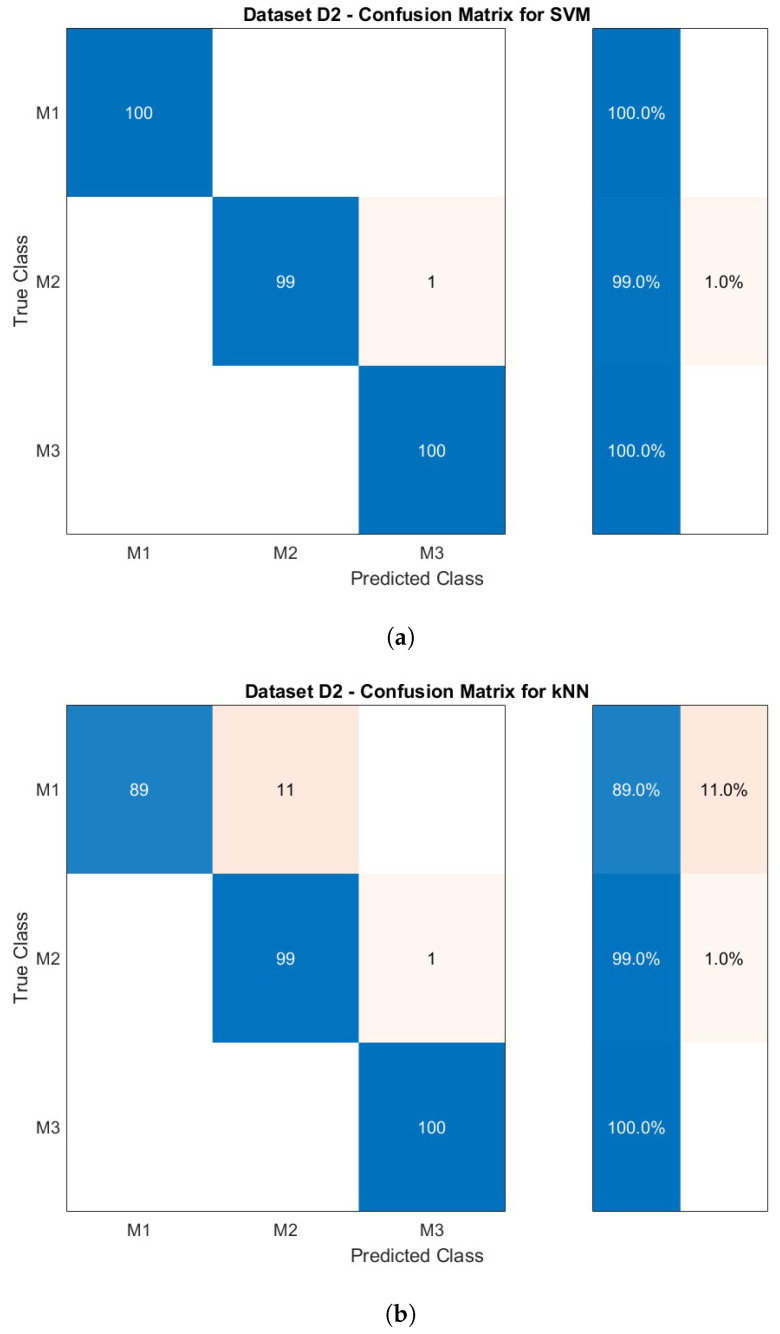
Confusion matrix for dataset D2 for classifier SVM (**a**) and classifier kNN (**b**) along with the sensitivity on the right side (blue).

Two supervised classification algorithms (SVM and kNN) were selected for comparison for this three-class classification problem with the features described in Table 2 used as input variables. Table 4 presents the classification results for the two datasets D1 and D2 compiled from the three experiments as summarised in Table 3. Further, the regularization parameter (*C*) for the SVM and the number of neighbours (*k*) for the kNN that provide the best performances are shown. The confusion matrix for dataset D2 is visualised in Figure 6 along with the sensitivity. The matrix is used to identify which materials are misclassified and account for a decrease in the overall accuracy. Moreover, the confusion matrix is essential to interpret the obtained results because it allows us to identify which materials are frequently confused by the classifiers.

For dataset D1 with all three experiments present in the training set and the unseen data from the experiments forming the testing set, both classifiers yield 100% of accuracy. Considering the observations made in Figure 4 and Figure 5, this very good result appears plausible. Dataset D2 was used to assess if the specific signal dynamics of one material could be sufficiently captured using Exp. 1 and Exp. 3 for training. They were selected because both experiments represent the maximum disparity of palpation parameters for signal acquisition. The unseen data acquired in Exp. 2 was used as a testing set. While the overall accuracy drops slightly to 99.67% for SVM and 96.00% for kNN, both results appear very good for the given scenario. This shows that even if different parameters of the palpation process change, the signal behaviour has the power to differentiate the materials.

Figure 6 shows the confusion matrix for dataset D2 for both classifiers along with the sensitivity for the materials on the right side (blue). Given the overall accuracy for SVM, the single misclassification of $M_{2}$ could possibly be an outlier. A similar observation for $M_{2}$ can be made for the kNN. In addition, the drop in the overall accuracy of the classification is caused by a misclassification of 11 signals from $M_{1}$ as $M_{2}$.

## 4. Discussion

### 4.1. Classification

For dataset D1, both classifiers showed a perfect classification performance. This result illustrates the significance of the extracted features from the palpation signals for the individual materials; 70 signals per material and experiment result in (a sufficient) 210 signals per material and appear to be prerequisites for this performance. Further, sufficient variability in the training data is given by the differences in the palpation parameters per experiment. This reflects in the exemplary features shown in Figure 5. From the 3 visualised features, $SE_{HF}$ appears to be important for distinguishing the materials. For an assessment of the individual features and their contribution to distinguishing the materials, feature ranking should be performed in future studies. However, based on the visible formation of easily distinguishable clusters per material in Figure 5, the good classification results confirm this qualitative impression. Another explanation for the good performance is the limited number of classes along with the considerable differences in the materials. The surfaces of $M_{1-3}$ differ clearly in terms of smoothness and surface irregularities. For example, materials 1 and 2 are characterised by irregular surfaces, namely the fibre structure of the textile felt and the studs of the rubber material. These result in recurrent small excitations or bumps in the case of material 2, which translate into comparably low-frequency components in the palpation signals. In contrast, the uniform surface of the foamed plastic material 3 causes an interaction mainly characterised by friction. This results in higher frequency components of the palpation signal that are visually reflected in the CWT spectrum and can be quantified by high values of $SE_{HF}$. The uniform linear palpation with stable indentation depth *z* leads to substantial differences in the interaction experienced by the palpation instrument. These differences are similarly reflected in the palpation signal acquired and can be sufficiently covered by the extracted features.

In a realistic application scenario, in MIS, the parameters for palpation cannot always be perfectly controlled and defined; thus, Dataset D2 was analysed. By comparison, for D2, the accuracy for SVM decreases only marginally, while the kNN provides a comparably poor result with very good (≈96%) accuracy. The minimal drop in accuracy for SVM is from a single misclassification of $M_{2}$, as can be seen in Figure 6a. A similar misclassification of one palpation signal from $M_{2}$ can be observed for the kNN in Figure 6b. A verification of the data showed that the same signal was misclassified in both cases. This suggests that the respective signal might be an outlier from the dataset for $M_{2}$. The performance of the SVM shows that the training data from Exp. 1 and Exp. 2 with the highest variety of the palpation parameters α and $\vec{v}$ allows for a good generalisation of the classifier. As a result, the classification performances for the previously unseen palpation signals from Exp. 2 are satisfying. The changes in the palpation parameters α and $\vec{v}$ for the acquisition appear to be irrelevant for the correct classification in this case.

In contrast, the noticeable drop in overall accuracy for the kNN is caused by additional misclassifications of 11 signals from $M_{1}$ as $M_{2}$. A possible explanation for this drop is the hyperparameter optimisation for the number of neighbours *k* performed on the training data. The optimisation based on the spatial distribution in the multi-dimensional feature space led to a number of k=45 neighbours to be considered for the classification of an individual palpation signal. With respect to the 200 signals per material involved in the training, this appears to be a comparably high number. Further, the absence of palpation signals from Exp. 2 in the training data seems to promote a bias of the classifier. Considering Figure 5, this particularly reflects in material $M_{1}$ and $M_{2}$, where the inclusion of data from Exp. 2 would have had a positive effect on the variance in the training data. This observation is supported by the fact that for the testing dataset, only the classification of material $M_{1}$ is affected.

In addition, based on the exemplary features visualised in Figure 5 it seems that the distributions of materials $M_{1}$ and $M_{2}$ are spatially closer in the feature space compared to $M_{3}$. This might also hold true for the other features or the material’s representation in the multi-dimensional feature space in general. The evolved bias, the high number of neighbours and the spatial proximity of $M_{1}$ and $M_{2}$ add to the problem and appear to promote a misclassification of $M_{1}$ in this case. Given an unchanged compilation of the dataset, a potential solution could be a limitation of the number of neighbours *k* during the hyperparameter tuning. Another approach could be a change in the used distance metric from the Euclidean distance to a weighted metric, such as weighted Euclidean distance.

The proposed signal processing approach appears suitable to extract a number of features that capture the individual signature of the palpation signal. The comparably simple features allow for the reliable discrimination of the materials utilising standard classification algorithms. Regarding application in MIS (and more realistic material samples), additional features capturing the smoothness, stability, and degree of the complexity of a signal should be explored. In summary, while both classifiers provide very good performances, SVM appears to be preferable over the kNN in this particular application.

### 4.2. Experimental Setup

The experimental setup was designed to assess the influence of parameters such as contact angle or palpation velocity on the vibro-acoustic signals originating from this interaction. However, it comes with a number of limitations related to this purpose as well as related to the application in a realistic scenario of MIS. While it allows for defined and stable values for the parameters α, $\vec{v}$, and the position of the instrument tip in the 3D space, a substantial drawback is the absence of a force measurement. Due to the constant indentation depth *z* throughout the experiments and the individual mechanical properties of the materials, the contact forces between the instrument tip and material differed per experiment and material. Further, the highly different surfaces led to different surface areas of contact and with that friction forces. While the FCI gives access to estimations of the external torques obtained from the internal joint torques, the reliable measurement of contact forces is not feasible with the experimental setup used. The integration of a proprietary force sensor into the end-effector comprising the vibration measurement system could solve this problem and allow for an accurate setting of contact force. A method for the measurement of interaction forces as presented in [46] could be integrated into the experiment for this purpose. With respect to an application in MIS, this would be crucial to limit the maximum force exerted on the tissue or organ.

Moreover, the influence of the instrument used for the palpation needs to be discussed. Geometrical parameters, such as the shape and dimension of the tip in contact have an influence on the acquired signal. For a given rough surface, a smaller and more pointed tip will produce more vibrations and thus excitations in the signal than a bigger and rounded tip. A pointed tip allows for more interactions and collisions with the micro-structure of a surface while a rounded tip has some form of averaging effect. Further, the contact surface area and with that friction changes for an identical indentation depth based on the size and shape of the tip. For medical palpation probes, such as the one used in this study, mainly rounded tips are used to prevent any damage or even rupture of the tissue palpated. The dimensions—especially the diameters of such probes—vary to a limited extent due to handling and stability reasons. In addition to these geometrical aspects, the mechanical properties of the instrument do have an influence on the vibrations originating from the interaction and their propagation. For medical instruments typically stainless steel is used as the base material. Individual variations in the composition and mechanical properties of the material presumably influence the intensity of the acquired vibration signal. Because of this, only one specific instrument should be used for the acquisition of the palpation signals for a particular purpose or study. However, the influence of the mentioned geometrical and mechanical characteristics of the palpation instrument should be assessed in an individual study. Further, it should be checked whether the signals acquired with different instruments for a given palpation interaction are comparable. Or if they contain similar information that can be extracted in form of features.

Noticeable limitations of the presented study are the substantial differences in the palpated materials. To assess the influence of the palpation parameters α and $\vec{v}$ for a broad range of material characteristics, it appears logical to use a set of sample materials with varying mechanical and surface properties. However, the results of the classification show that the selected materials are maybe too distinct in their characteristics. The surface texture of $M_{2}$ presents an extreme case in this sense. The rubber studs of the material cause an alternation between short segments of friction due to the palpation and transient excitations due to the collisions with the studs. This behaviour is clearly reflected in the acquired signals, as can be seen in Figure 4b. However, in the medical field, for palpation-based differentiation of textures, the surfaces would presumably be substantially similar in many aspects. Therefore, in cases of low-frequency components, processing strategies and features with better resolution in low-frequency ranges would be required. Compared to the materials used in this study, the biological tissues and organs to be differentiated are much closer in terms of composition, surface structure, and mechanical behaviour. Moreover, in the given scenario of MIS all examined tissues would most likely experience any form of lubrication due to the presence of body fluids. A study using a selection of realistic materials is necessary to investigate the influence of the proposed approach and the classification performance. However, for some medical applications, the similarity between the soft tissue surfaces to be distinguished might not be as crucial. One example is the field of orthopaedic surgery, where the differentiation of structures such as bone, ligaments, or cartilage might be of interest. In addition to an application in arthroscopy, such as typical MIS, an application in conventional open surgery, such as joint replacement, is imaginable.

While the experimental setup illustrates the concept of robot-assisted palpation, certain MIS-specific conditions were neglected. Vibrations originating from the actuation of the robot were not noticeable in the acquired palpation signals. An explanation could be that the energy of these vibrations was significantly smaller compared to the interaction vibrations. Alternatively, the vibrations could be located in a different frequency band above the 7 kHZ considered in this study. However, in MIS surgical instruments are usually introduced to the body via port systems, such as a cannula or trocar. A related interaction of the instrument with such systems will certainly cause disturbing vibrations that need to be considered in the processing step. Further, the port acts as a pivot point for the movement of the instrument inside the body. Thus, the range of motion and the potential palpation path is limited. This needs to be considered in the path planning for the robotic actuation. The mentioned limitations need to be addressed in a future study with help of a realistic phantom comprising the typical setup of MIS.

### 4.3. Influence of Contact Angle and Palpation Velocity

Changes in the two investigated parameters α and $\vec{v}$ have been found to have differing effects on the palpation signals. As it is easy to observe from Figure 4, a doubling of $\vec{v}$ from Exp. 1 to 2 only has a marginal influence on the spectral signature in the CWT-based spectrum. This reflects as well in the averaged spectrum, where only small increases of energy following the morphology of the curve are noticeable. For materials $M_{1}$ and $M_{3}$, this can be explained by the smooth surface of the material. The increase in velocity mainly has an influence on kinetic friction. However, it remains stable during the palpation process, which leads to a largely stationary signal. Material $M_{2}$ shows similar behaviour in terms of increasing energy in the averaged spectrum. However, the level of energy increase is much bigger compared to $M_{1}$ and $M_{3}$. This difference can be explained by the rubber studs causing recurring collisions with the instrument. A higher velocity results in an increase in the energy of these excitations. Thus, the collisions are easy to locate in the CWT-spectrum compared to Exp. 1 and cause a bigger increase of energy in the averaged spectrum. Having said that, the CWT-based signature per material is little influenced by the velocity. The effect on the IDF appears to be bigger. While the IDF’s variance increases with $\vec{v}$ for $M_{1}$ and $M_{3}$, it shows the opposite behaviour for $M_{2}$. For the first, the increase might be explained by the continuous collisions on the microscopic level that characterise a friction interaction. The higher velocity causes an increase of energy of the collisions and with that more variety of the IDF. For $M_{2}$, the IDF’s behaviour shows more stationary segments. This might be explained by a declining excitation between the collisions. According to these observations, features derived from the IDF might be better suited to detect a change in the palpation velocity.

Compared to $\vec{v}$, the contact angle appears to have a bigger influence on the energy of the palpation signal. For all materials, the change to $\alpha =70^{\circ }$ results in a significant increase of energy in the signal. This increase can be explained by the force decomposition depicted in Figure 1b along with Equation (1). The steeper contact angle of $70^{\circ }$ causes an increase in the axial force component ${\vec{F}}_{axial}$ acting on the instrument. This results in a stronger excitation in the axial direction that is captured by the axially placed membrane on the opposite end. Besides the noticed change in the amount of energy, the general characteristic of the CWT-based signature remains consistent. In the case of α, the IDF’s behaviour appears to be less influenced compared to $\vec{v}$. Accordingly, features derived from the CWT-based (stationary) spectrum seem to be better suited to detect changes in the contact angle.

In general, the investigated parameters appear to have an acceptable influence on the palpation signals when staying within certain limits. This can be confirmed by the good results for the classification using dataset D2. This is an important criterion for a future application in MIS. Admittedly, the number of experiments and with that different combinations and values for α and $\vec{v}$ was small. A repetition of the study with a wider range of values seems reasonable. Even a random generation of parameter values and combinations would be feasible due to the ease of control and the adaptability of the robotic palpation.

## 5. Conclusions

In summary, it is possible to extract information in the form of features from the specific time–frequency signature of the palpation signals acquired with a simple vibration measurement system. The signature retains its general characteristic even if the conditions of the palpation change within certain limits, but is highly dependent on the interacting material. It allows for a differentiation of materials based on the extracted features, even with a not perfectly controlled experimental setup and parameters of palpation. This robustness of the presented approach is a prerequisite for any realistic application in MIS. This especially accounts for the intended application in robotic palpations, where the specific requirements and constraints of the surgical environment do not always allow identical palpation parameters. How the extracted information from the signals should be presented is still an open question. In general, a presentation as visual, haptic, or acoustical information is possible. The direct mapping of the vibrations to an actuation mechanism is as conceivable as a parameterization of interaction events and presentation as acoustic feedback. Even a use-as-directed control signal for semi-autonomous surgical systems seems possible.

For the first application in robot-assisted surgery, the integration of the sensing concept into an already existing and regularly performed robot-assisted procedure would be beneficial. Ideally, the procedure should be highly specific, with limited variation in the palpated tissue or surface, and with a strict workflow and sequence of actions. One such procedure could be the robot-assisted replacement of a knee joint, known as arthroplasty. Several robotic systems, including Mako SmartRobotics (Stryker Corporation, Kalamazoo, MI, USA) [47] and the ROSA^®^ Knee System (Zimmer Biomet Holdings, Inc., Warsaw, IN, USA) [48] are already established in clinical practice for this procedure. The surgical planning in robot-assisted arthroplasty is based on a combination of preoperative imaging and intraoperative identification and tagging of surgical landmarks. Because it is a conventional open surgery, some of the limitations related to the experimental setup as pointed out earlier do not apply in this case. A robot-assisted palpation of the tissue surface intended to be replaced could potentially complement the planning process. It could intraoperatively provide valuable information regarding the condition of the tissue or its surface. Such information would be of high value for the identification and grading of osteoarthritic cartilage and the support of treatment decision between a partial or a full knee replacement. In a similar way, arthroscopic procedures could benefit from this approach in combination with robotic assistance [49,50].

## Figures and Tables

**Table 1:** Summary of parameters for experiments 1–3. For the acquisition of signals $S_{n=1-100}$ the velocities ${\vec{v}}_{y}={\vec{v}}_{z}=0$ were kept constant during the palpation phase for all three experiments. The *z* coordinate of the robotic arm and the height of the materials on the table were kept constant.

Experiment	Palpation Parameter				Signal
	α	${\vec{v}}_{x}$	Length	Path	
Exp. 1	$30^{\circ }$	33.3mm/s	0.75 s	25 mm	$S_{n}(M,\;30^{\circ },\;33.3\;$ mm/s)
Exp. 2	$30^{\circ }$	66.6 mm/s	0.75 s	50 mm	$S_{n}(M,\;30^{\circ },\;66.6\;$mm/s)
Exp. 3	$70^{\circ }$	66.6 mm/s	0.75 s	50 mm	$S_{n}(M,\;70^{\circ },\;66.6\;$ mm/s)

**Table 2:** Summary of the energy-related and statistical features derived from the computed indicators.

Nr.	Type	Computation Basis	Name	Description
1	energy related	CWT-based Stationary Spectrum	TE	total energy of the spectrum
2			$SE_{VLF}$	energy in the VLF band
3			$SE_{LF}$	energy in the LF band
4			$SE_{MF}$	energy in the MF band
5			$SE_{HF}$	energy in the HF band
6		IDF’s Histogram	$HE_{VLF}$	histogram’s energy in VLF band
7			$HE_{LF}$	histogram’s energy in the LF band
8			$HE_{MF}$	histogram’s energy in the MF band
9			$HE_{HF}$	histogram’s energy in the HF band
10	statistical	CWT Spectrum	$F_{max}$	frequency of maximum excitation
11		IDF	$DF_{max}$	maximal dominant frequency
12			$DF_{min}$	minimal dominant frequency
13			$DF_{var}$	variance of dominant frequency

**Table 3:** Summary of the composition of training and testing set for datasets D1 and D2. Per material, D1 results in 210 signals for training and 90 signals for testing and D2 results in 200 and 100 signals for training and testing.

Dataset	Training Set	Testing Set
** D1 **	70% of $S_{n}(M,\;30^{\circ },\;33.3\;$mm/s)	30% of $S_{n}(M,\;30^{\circ },\;33.3\;$mm/s)
	70% of $S_{n}(M,\;30^{\circ },\;66.6\;$mm/s)	30% of $S_{n}(M,\;30^{\circ },\;66.6\;$mm/s)
	70% of $S_{n}(M,\;70^{\circ },\;66.6\;$mm/s)	30% of $S_{n}(M,\;70^{\circ },\;66.6\;$mm/s)
** D2 **	$S_{n}(M,\;30^{\circ },\;33.3\;$mm/s)	$S_{n}(M,\;30^{\circ },\;66.6\;$mm/s)
	$S_{n}(M,\;70^{\circ },\;66.6\;$ mm/s)	

**Table 4:** Classification results for datasets D1 and D2 for the optimised parameters *C* and *k*.

Dataset	Classifier		Accuracy	Material	Sensitivity	Precision	$F_{1}$ score
D1	SVM	C=1	1	$M_{1-3}$	1	1	1
	kNN	k=46	1	$M_{1-3}$	1	1	1
** D2 **	SVM	C=1	0.9967	$M_{1}$	1	1	1
				$M_{2}$	0.9900	1	0.9950
				$M_{3}$	1	0.9901	0.9950
	kNN	k=45	0.9600	$M_{1}$	0.8900	1	0.9418
				$M_{2}$	0.9900	0.9000	0.9429
				$M_{3}$	1	0.9901	0.9950

## Data Availability

Restrictions apply to the availability of these data. Data was obtained from SURAG Medical GmbH and are available from the authors with the permission of SURAG Medical GmbH.

## Abbreviations

The following abbreviations are used in this manuscript:

MIS	minimally invasive surgery
RMIS	robot-assisted minimally invasive surgery
FCI	Franka control interface
ROS	robot operating system
CWT	continuous wavelet transform
DWT	discrete wavelet transform
SVM	linear support vector machine
kNN	k-nearest neighbours
VLF	very low-frequency sub-band
LF	low-frequency sub-band
MF	middle-frequency sub-band
HF	high-frequency sub-band
DF	dominant frequency
IDF	instantaneous dominant frequency
$F_{max}$	frequency of maximal excitation
TE	total energy of the spectrum
$SE_{VLF-HF}$	spectral energy of sub-band
$HE_{VLF-HF}$	IDF’s histogram energy of sub-band
$M_{1-3}$	material 1–3
D1, D2	Dataset 1, Dataset 2
α	contact angle
$\vec{v}$	palpation velocity
